# Outcome and long-term follow-up of adrenal lesions in multiple endocrine neoplasia type 1

**DOI:** 10.20945/2359-3997000000170

**Published:** 2019-08-28

**Authors:** Mara Ventura, Miguel Melo, Francisco Carrilho

**Affiliations:** 1 Universidade da Beira Interior Faculty of Health Sciences University of Beira Interior Covilhã Portugal Faculty of Health Sciences, University of Beira Interior, Covilhã, Portugal; 2 Endocrinology Diabetes and Metabolism Department University and Hospital Center of Coimbra Coimbra Portugal Endocrinology, Diabetes and Metabolism Department, University and Hospital Center of Coimbra, Coimbra, Portugal; 3 Universidade de Coimbra Faculty of Medicine University of Coimbra Coimbra Portugal Faculty of Medicine of the University of Coimbra, Coimbra, Portugal

**Keywords:** Multiple endocrine neoplasia type 1, adrenal glands, autonomous cortisol secretion

## Abstract

**Objective:**

To describe the prevalence, clinical characteristics and outcome of adrenal lesions in long-term follow-up of Multiple endocrine neoplasia type 1 (MEN1) patients.

**Subjects and methods:**

We retrospectively studied sixteen patients from six families of individuals with MEN1. Adrenal involvement was evaluated using clinical, biochemical and imaging data.

**Results:**

Adrenal lesions were identified in nine of sixteen (56.3%) patients: seven women and two men (mean age: 52.2 years). Adrenal involvement was detected at MEN1 diagnosis in more than half of the patients. Eighteen adrenal nodules were founded (median of two nodules per patient) with mean adrenal lesion diameter of 17.4 mm. Three patients had unilateral adrenal involvement. Hormonal hypersecretion (autonomous cortisol secretion) was found in two patients. None of the patients was submitted to adrenalectomy, presented an aldosterone-secreting lesion, a pheochromocytoma, an adrenal carcinoma or metastatic disease during the follow-up. A predominance of stable adrenal disease, in terms of size and hormonal secretion, was observed. Adrenal lesions were evenly distributed between the germline mutations.

**Conclusion:**

Adrenal tumours are a common feature of MEN1 that can affect more than half of the patients. Most of the tumours are bilateral non-functional lesions, but hormonal secretion may occur and should be promptly identified to reduce the morbidity/mortality of the syndrome. Periodic surveillance of these patients should be performed.

## INTRODUCTION

Multiple endocrine neoplasia type 1 (MEN1) is a disorder characterized by the occurrence of tumours in two or more endocrine glands of a patient. It is a rare disease, with an estimated prevalence of one in 30 000 individuals, with a high penetrance and an equal sex distribution (1). It is inherited as an autosomal dominant disease caused by mutations or cytogenetic alterations involving the *MEN1* gene, which is a tumour suppressor gene located on chromosome 11q13 that encodes for a protein named menin (2).

MEN1 syndrome is typically characterized by the occurrence of primary tumours of the parathyroid glands (95% of the patients), endocrine pancreas (30-80% of the patients) and anterior pituitary gland (15-90% of the patients) (1). Other endocrine and non-endocrine tumours, such as adrenal lesions, bronchial carcinoids, gastrointestinal tract carcinoids, thymus neoplasms, lipomas, angiofibromas and colagenomas, have also been described in variable frequencies (3). This syndrome may affect all age groups and more than 95% of the patients present manifestations of the disorder by the age of 50 years (4).

Adrenal lesions have been reported in about 36-73% of MEN1 patients (5-8). They are often diagnosed as an incidentaloma during radiological workup and most of them are non-functional lesions. Previous studies have reported a much lower frequency of adrenal lesions in the general population: around 5-10% (9).

The purpose of this study was to describe the prevalence, clinical characteristics and long-term outcome of adrenal lesions in a cohort of sixteen patients from six different MEN1 families.

## SUBJECTS AND METHODS

We retrospectively evaluated patients with genetically confirmed MEN1, diagnosed and followed in our department between 1 January 1995 and 31 December 2017. Patients were selected if specific adrenal imaging - computed tomography or magnetic resonance imaging – had been performed during their follow-up. When a nodule was identified, the following imaging characteristics were considered: size, density (Hounsfield units), margins and washout of the contrast media. In our cohort, the diagnoses of adrenal lesions were made by adrenal computed tomography (CT) during the routine screening of MEN1 patients, together with corresponding biochemical data. These patients were submitted first to an abdominal CT scan during the assessment and screening for adrenal and pancreatic lesions; after the identification of adrenal lesions, the patients were rescanned with an adrenal CT for better characterization. In patients who performed more than one CT, we selected the scan closest to the date of diagnosis and, in order to evaluate adrenal lesions progression during the follow-up, we selected the most recent available CT scan. In all patients, contrast medium was injected intravenously during the CT scan.

For patients with adrenal nodules, the following endocrine evaluation was performed during hospital admission: low-dose dexamethasone suppression test (LDDST: dexamethasone 1 mg orally between 23 and 24 p.m.), midnight serum cortisol, 24-hour urinary free cortisol and midnight salivary cortisol. Plasmatic aldosterone and renin samples were obtained in 89% of the patients with adrenal lesions (n = 8), after suspension and/or substitution of drugs that can affect the renin-angiotensin-aldosterone system and under standardized conditions. Plasmatic metanephrines were obtained in 78% of the patients (n = 7) and 24-hour urinary metanephrines were obtained in 67% of the patients (n = 6); five patients had both measurements in their clinical records. Plasma 17-hydroxyprogesterone concentrations were measured in 50% of the patients with bilateral adrenal lesions (n = 3).

We also considered clinical and demographic features of the included patients, namely age at diagnosis of MEN1, age at diagnosis of the adrenal lesion, gender and time elapsed since the diagnosis of MEN1 until the detection of the adrenal lesion. All the patients had a molecular alteration in *MEN1* gene consistent with the diagnosis of MEN1 syndrome and their genotype is reported using the reference sequence NM_130803. We collected all the data from the patients’ clinical records and from electronic hospital database. At diagnosis and then annually, patients underwent clinical, biochemical and radiological evaluation, with special regard to symptoms known to be associated with hormone hypersecretion. We collected all the data closest to the moment of diagnosis and, to evaluate patients’ follow-up, we collected the most recent available data. All the patients signed an informed consent approved by the Internal Reviewing Board, and all the procedures described in this study were in accordance with national and institutional ethical standards.

## RESULTS

Table 1 shows the demographics and clinical diagnosis of the MEN1 patients with adrenal lesions.

**Table 1:** Patients’ demographics, diagnosis and biochemical data

Patient	Gender, age at MEN1 diagnosis	Age at diagnosis of adrenal lesions	Years elapsed since MEN1 diagnosis until adrenal lesions detection	Diagnoses	Adrenal lesions (mm)		Cortisol				Aldosterone / renin ratio (ng/dL per ng/mL/h)	Metanephrines (metanephrine/ normetanephrine)		17-OHP (ng/mL)
	
					Right	Left	LDDST (µg/dL)	Midnight serum cortisol (µg/dL)	UFC (µg/24h)	Midnight salivary cortisol (µg/dL)		Plasmatic (pg/mL)	Urinary (µg/24h)	
1	F, 50	53	3	PHPT, pancreatic NET, NFPA, lung carcinoid	9.0	16.0	1.0	6.7	57.0	NP	22.0	NP	NP	NP
2	F, 51	51	0	None	NA	15.0	1.2	NP	58.0	0.8	16.5	38.1/47.2	NP	NP
3	M, 40	58	18	PHPT, pancreatic NET, acromegaly	15.0	25.0	1.5	3.2	64.0	NP	5.0	20.9/22.4	100.7/333.6	1.1
4	F, 18	34	16	PHPT, pancreatic NET	15.0, 13.0	24.0, 14.0, 11.0	14.0	7.3	97.0	0.4	17.4	32.6/60.5	66.5/285.9	2.8
5	M, 40	40	0	PHPT, insulinoma, NFPA	30.0	20.0	NP	6.5	158.0	NP	NP	NP	0.2/NP	NP
6	F, 40	56	16	PHPT, gastrinoma, acromegaly	11.0	14.0	< 1.0	3.2	33.0	0.1	9.9	57.1/61.8	95.6/189.4	0.24
7	F, 68	68	0	Atypical thymic carcinoid, pancreatic NET, PHPT	19.0	16.0	2.1	6.6	34.0	NP	5.5	28*/392**	30.1/NP	NP
8	F, 57	57	0	PHPT	NA	10.0	NP	3.7	NP	NP	322.4	18.8/36.0	61.8/83.8	NP
9	F, 53	53	0	Typical bronchial carcinoid, PHPT, pancreatic NET	36.0	NA	5.9	7.6	30.0	NP	12.8	31.1/35.3	NP	NP

F: feminine; M: masculine; NP: not performed; NA: not applicable; LDDST: low dose dexamethasone suppression test (normal range: ≤ 1.8 µg/dL); UFC: urinary free cortisol (normal range: 10-80 µg/24h); 17-OHP: 17-hydroxydeprogesterone (normal range: 0.2-4.7 ng/mL); NET: neuroendocrine tumour; PHPT: primary hyperparathyroidism; NFPA: non-functioning pituitary adenoma. Midnight serum cortisol normal range: ≤ 1.8 µg/dL; midnight salivary cortisol normal range: < 0.1 µg/dL; aldosterone/renin ratio normal range: < 25; plasmatic metanephrines normal range: < 60 pg/mL, plasmatic normetanephrines normal range: < 120 pg/mL; urinary metanephrines normal range: 30-350 µg/24h; urinary normetanephrines normal range: 50-650 µg/24h. *Altered plasmatic metanephrine normal range: < 125 pg/mL. **Altered plasmatic normetanephrine normal range: < 600 pg/mL.

We evaluated a total of sixteen patients with genetically confirmed MEN1. The group includes nine women and seven men with a mean age of 35.9 years (12-68 years) at MEN1 diagnosis. Mean follow-up was 126 months (8-324), which corresponds to approximately 11 years. Adrenal lesions were identified so far in nine of these patients (56.2%), seven women and two men, with a total of eighteen adrenal lesions and a median of two lesions per patient. These nine patients are distributed in five different families (family A: patients 1 and 2; family B: patients 3 and 4; family C: patients 5 and 6; family D: patient 7; family E: patients 8 and 9). The average patients’ age at diagnosis of adrenal lesions was 52.2 years (34-68 years), approximately 6 years after MEN1 diagnosis (up to 18 years) and more than half of the lesions were detected at MEN1 diagnosis.

### Radiologic characteristics of adrenal lesions

The mean adrenal lesion diameter at diagnosis was 17.4 mm (9-36 mm), with most of the lesions (78%) being 20 mm diameter or smaller, and most of the patients (67%) had bilateral adrenal involvement. All the adrenal lesions identified had less than 10 Hounsfield units on CT scan and had a fast washout of the contrast agent.

**Figure 1:**
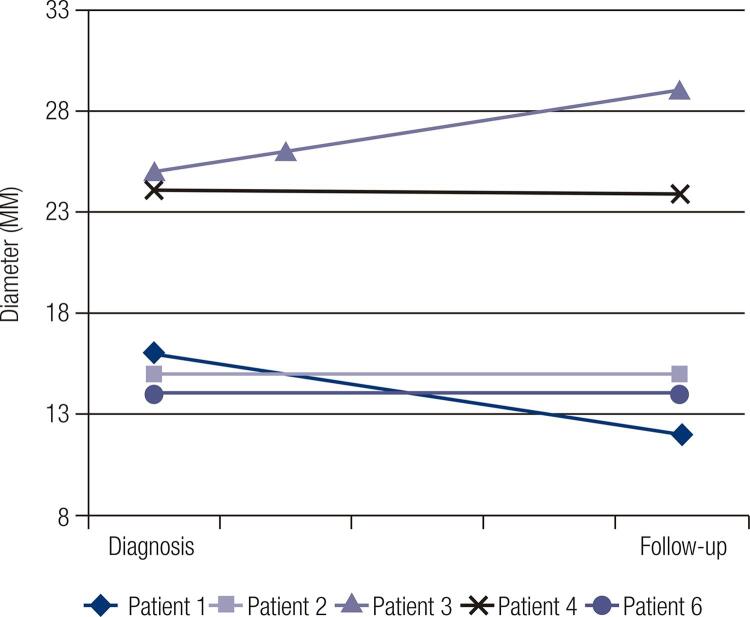
Evolution of left adrenal lesions diameter.

**Figure 2:**
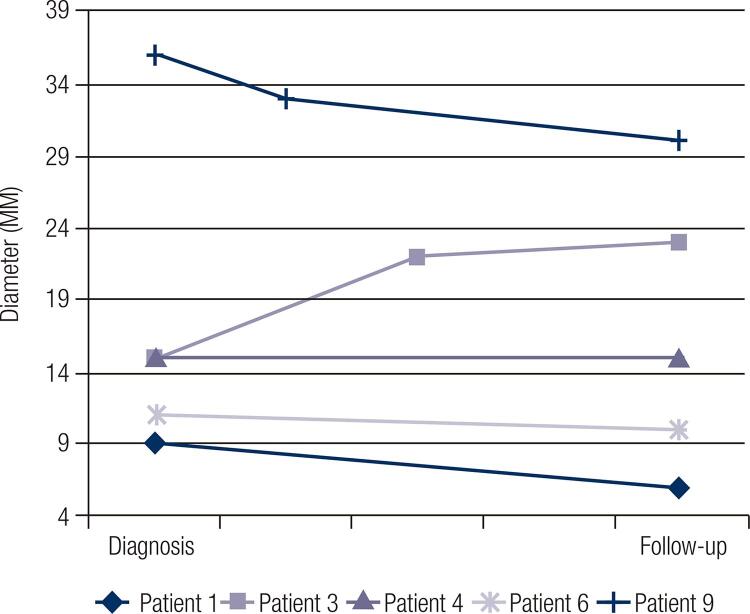
Evolution of right adrenal lesions diameter.

Figures 1 and 2 show the size evolution of adrenal lesions during the follow-up. Patients 5, 7 and 8 only have available information about adrenal lesion diameter at the diagnosis and, as so, were not included in the mentioned figures: patient 5 was diagnosed in our Department but was subsequently followed in another hospital due to personal reasons; patient 7 died before performing a second adrenal evaluation and patient 8 has recently been diagnosed and has not yet perform additional imaging evaluation. In our cohort, most of the patients presented a stabilization or even a slight decrease in adrenal lesions diameter (Figures 1 and 2). In fact, only patient 3 presented an increase of approximately 16% of left adrenal lesion diameter and 53% of right adrenal lesion diameter; nevertheless, recent data demonstrate that those are non-functional adrenal lesions. Patient 4 and 9, which had autonomous cortisol secretion, did not show an increase in adrenal lesions size and patient 9 presented actually a 17% decrease in adrenal lesion diameter (from 36 mm to 30 mm) according to the last radiologic evaluation.

### Functional characterization of adrenal lesions

Seven patients had non-functional adrenal lesions. Autonomous cortisol secretion was founded in two patients (patients number 4 and 9), belonging to different families. Patient 4 is a female that has bilateral adrenal lesions and is the patient of our cohort with the highest number of adrenal nodules (n = 5), with an average diameter of 15.4 mm (11-24 mm); in this patient, adrenal lesions where identified about sixteen years after MEN1 diagnosis. Patient 9 is also a female that has only one adrenal lesion with 36 mm, which corresponds to the largest adrenal lesion in your sample, and it was identified at the time of MEN1 diagnosis. These two patients didn’t have clinical signs of overt Cushing’s syndrome but their serum cortisol levels after 1 mg dexamethasone were above 5 µg/dL. In addition, patient 4 also had a 24-hour urinary free cortisol (UFC) above the normal range values, and she had osteoporosis of the lumbar spine with an increased fracture risk. Patient 9 had no comorbidities potentially related to autonomous cortisol secretion. The remaining adrenal lesions were non-functioning. Patient 8 presented an increased aldosterone/renin plasmatic ratio; she performed a saline infusion test that excluded primary hyperaldosteronism. None of the nine patients was submitted to adrenalectomy; the two patients with hormonal hypersecretion were submitted to neither surgical nor pharmacological management. Patient number 5 doesn’t have additional biochemical data because after the diagnosis he was subsequently followed in another hospital. According to the available data, he died of unknown cause ten years after the diagnosis. Patient number 7 died four years after the diagnosis, due to respiratory complications related to pulmonary metastases from atypical thymic carcinoid.

During the follow-up, patients 4 and 9 presented a cortisol level reduction to the level of “possible autonomous cortisol secretion” on low dose dexamethasone suppression test: the cortisol level of patient 4 decreased to 3.12 µg/dL and to 3.20 µg/dL in patient 9. No clinical or biochemical evidence of hormonal secretion was found in the remaining patients.

### Molecular genetics of MEN1 syndrome

Adrenal lesions were evenly distributed between the different germline mutations (Table 2).

**Table 2:** MEN1 germline mutations and adrenal lesions identified. The reference sequence used for the nomenclature was NM_13080

Patient	Gender, age at diagnosis of adrenal lesions	Number of adrenal lesions	Adrenal lesions diameter (mm)	Bilateral lesions	Hormonal hypersecretion	Mutation	Protein impact	Clinical significance
**1**	F, 53	2.0	9.0-16.0	yes	no no	c.1A>T (p.Met1Leu)	Missense	Pathogenic (21,22)
**2**	F, 51	1.0	15.0	no				
**3**	M, 58	2.0	15.0-25.0	yes	no	c.1546delC (p.Arg516GlyfsX43)	Frameshift	Pathogenic (23,24)
**4**	F, 34	5.0	11.0-24.0	yes	yes			
**5**	M, 40	2.0	20.0-30.0	yes	no	c.628_631delACAG (p.Thr210SerfsX13)	Frameshift	Pathogenic (25,26)
**6**	F, 56	2.0	11.0-14.0	yes	no			
**7**	F, 68	2.0	16.0-19.0	yes	no	c.637delG (p.Ala213ProfsX11)	Frameshift	Likely Pathogenic (22,27)
**8**	F, 57	1.0	10.0	no	no	c.260_280del (p.Leu89_Ala95del)	In frame	Likely pathogenic (28)
**9**	F, 53	1.0	36.0	no	yes			

In our cohort, only one mutation (c.260_280del) was exclusively associated with unilateral adrenal involvement. Three mutations (c.1A>T, c.628_631delACAG and c.637delG) were associated with non-functional adrenal lesions in all the affected patients.

The seven patients alive remain under tight clinical, biochemical and imagiological surveillance.

### Associated endocrine tumours

Primary hyperparathyroidism (PHPT) represented the most common manifestation of MEN1 syndrome in our cohort, affecting 89% of the patients (n = 8). PHPT was the first manifestation of the syndrome in four patients and their median age of onset was 39 years (18-57). Subtotal parathyroidectomy was performed in all affected patients.

Pancreatic neuroendocrine tumours represent the second most frequent manifestation in our sample, affecting 76% of the patients (n = 7); most of them were non-functional and clinically silent pancreatic NET (n = 5). One insulinoma and one gastrinoma were identified in two different patients, and both were submitted to surgery.

Anterior pituitary tumours occurred in 44% of the patients (n = 4) and 50% of them secreted growth hormone.

Carcinoid tumours were identified in one third of the patients (n = 3): one patient had an atypical thymic carcinoid, one patient had a typical bronchial carcinoid and another patient had a lung carcinoid tumour.

One patient with a genetic MEN1 diagnoses did not have any clinical, biochemical or imagiological evidence of disease besides adrenal lesions (patient number 2).

**Figure 3:**
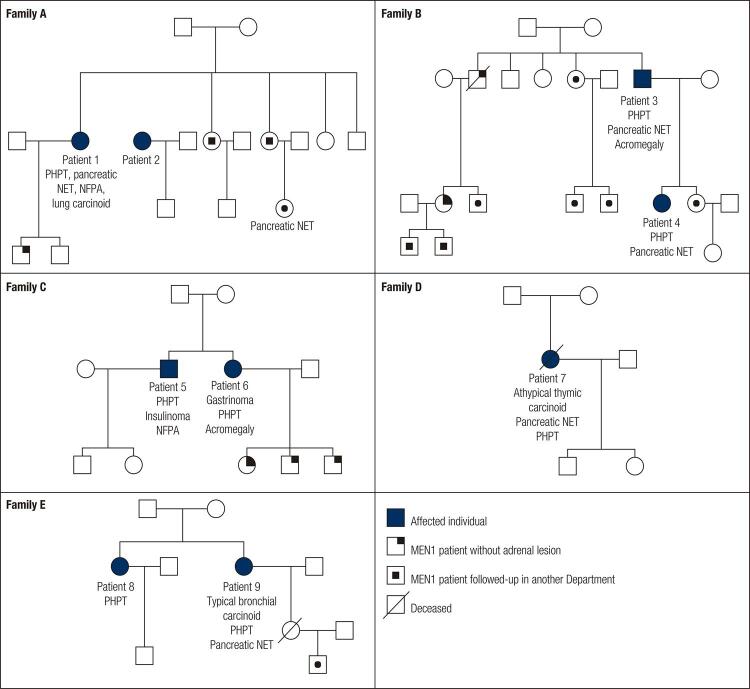
Genogram of MEN1 families of patients with adrenal lesions.

Figure 3 shows the family trees of the included patients, displaying all the clinical manifestations presented during follow-up.

## DISCUSSION

Adrenal lesions in MEN1 patients were first described in 1960s in autopsy studies or in laparotomies performed for other reasons (10). Some studies at that time reported a prevalence of adrenal lesions in MEN1 patients of around 9-45% (10). However, the lack of genetic tests to confirm the germline mutation left some patients out of these studies; as such, this prevalence reports are probably underestimated. After the availability of genetic tests, asymptomatic relatives of MEN1 patients were identified and screened for clinical, biochemical and radiologic manifestations of the disease, which increased the detection of adrenal lesions (11).

In the present study, adrenal lesions were identified six years after MEN1 diagnosis (0-18 years), which is in accordance with other case series (12); furthermore, more than half of the patients in our cohort had adrenal involvement, confirming previous series documenting that adrenal lesions are frequent in MEN1 syndrome and reinforcing that all patients with MEN1 should be screened for adrenal disease. On the other hand, patients with adrenal lesions should be evaluated for the possibility of MEN1 syndrome if they have additional characteristic features, although these lesions usually are not the first manifestation of the syndrome. In this cohort of MEN1 patients, most of the adrenal lesions were non-functional and only two patients had autonomous cortisol secretion, which confirms previously reported data (6,13-15). None of the patients of our cohort presented an aldosterone-secreting adrenal lesion, a pheochromocytoma, an adrenal carcinoma or metastatic disease during the study period. There was also no clinical evidence of the secretion of sexual steroids. Adrenal involvement was asymptomatic in all the patients in our cohort and these lesions were detected by a CT scan during the routine screening of MEN1 patients. In our sample, 67% of the patients had bilateral adrenal involvement, which is in accordance with previous studies (6). Besides their high frequency in MEN1 patients, consensus about the management of adrenal lesions as not yet been reached (14). In our cohort, only patient 4 may have formal indication for treatment because she had osteoporosis, a comorbidity potentially related to autonomous cortisol secretion. Nevertheless, this patient’s osteoporosis may have a multifactorial aetiology, as hyperparathyroidism may also have contributed to an early and severe bone mineral loss. Furthermore, several studies showed that bone mineral density improves in most patients after parathyroidectomy, even though this may take several months and may require transient calcium and calcitriol supplementation (16,17). Hypercortisolism resolution also proved to be associated with an improvement in bone mineralization (18,19). However, this patient refused any medical or surgical treatment for the adrenal lesions.

MEN1 syndrome is caused by germline mutations in *MEN1* gene that are evenly distributed throughout the gene, with less than ten reported mutations being found with a frequency above 1.5% (20). The genetic diversity of the syndrome is a barrier to establish clear genotype-phenotype correlations. Of note, in our study patients number 3 and 4, belonging to the same family and harbouring the c.1546delC mutation, had the higher number of adrenal lesions in this sample. Hormonal hypersecretion was found in two patients from different families, with different germline mutations: c.1546delC and c.260_280del. On the other hand, unilateral adrenal lesions were mainly restricted to patients harbouring the c.260_280del mutation. Although the small numbers preclude any definite conclusions, it would be interesting to evaluate if patients with MEN1 syndrome harbouring these germline mutations have higher frequency of adrenal nodules.

In conclusion, this study, performed on patients with genetically confirmed MEN1, demonstrates that adrenal lesions are a common feature of this syndrome regardless of their genotype. In our series, they occurred in more than half of the patients and were usually bilateral asymptomatic lesions that were detected approximately six years after MEN1 diagnosis by the radiologic work-up. Although a predominance of stable adrenal disease was observed in terms of size and hormonal secretion, some of them may cause hormonal hypersecretion and, as such, may be associated with higher morbidity and mortality and may contribute to patients’ impaired quality of life. Considering the variable prevalence of adrenal lesions reported by different authors and their potential to be hormonally active, adrenal evaluation should be considered in MEN1 patients; their prompt diagnosis would avoid delays and will enable an adequate treatment and follow-up of the affected patients. Moreover, we could not find any correlation between the germline mutation and the occurrence of adrenal lesions, which suggests that all patients with MEN1 syndrome should be evaluated for the presence of an adrenal lesion.

Prospective studies are necessary to strengthen our results, and to understand the global prevalence and impact of these lesions in patients’ outcome, in order to clarify and standardize their management and follow-up.
